# Comparison of efficacy and safety of 5-FU or capecitabine combined with cisplatin and docetaxel (mDCF and mDCX) as a first-line chemotherapy regimen in her 2-negative metastatic gastric cancer patients: A retrospective study

**DOI:** 10.1097/MD.0000000000037259

**Published:** 2024-03-01

**Authors:** Nebi Serkan Demirci, Abdulmunir Azizy, Nail Paksoy, İzzet Doğan, Senem Karabulut, Latif Karahan, Didem Tastekin

**Affiliations:** a Department of Medical Oncology, Cerrahpasa University Faculty of Medicine, Istanbul, Turkey; b Department of Medical Oncology, Istanbul University Institute of Oncology, Istanbul, Turkey; c Department of Medical Oncology, Tekirdağ Dr. Ismail Fehmi Cumalioğlu City Hospital, Tekirdağ, Turkey; d Department of Medical Oncology, Basaksehir Çam and Sakura City Hospital, Istanbul, Turkey; e Department of Medical Oncology, Hacettepe University Cancer Institute, Ankara, Turkey.

**Keywords:** advance disease, chemoterapy, gastric cancer, first line, fluoropyrimidines, mDCF, mDCX

## Abstract

The prognosis of metastatic gastric cancer (GC) is poor, with a median survival time of less than a year. Capecitabine is a prodrug, metabolized by thymidine phosphorylase to its cytotoxic metabolite (5-FU). Few studies have compared capecitabine and 5-FU in mGC. In this retrospective study, we compared the efficacy and safety of modified DCF (mDCF) (docetaxel, cisplatin, and 5-FU) and modified DCX (mDCX) (docetaxel, cisplatin, and capecitabine) regimens for first-line treatment in patients with mGC. The study included 112 mGC patients treated with either mDCF (n = 69) or mDCX (n = 43) between 2010 and 2021. Demographic data, response rate, progression-free survival (PFS), overall survival (OS), and adverse events were evaluated. The complete response rate in the mDCF group was 10.1%, whereas the complete response rate in the mDCX group was 2.3%. The partial response rate for mDCF and mDCX were 29% and 37%, respectively. The 2 treatment arms of the study had the same objective rate of response and disease control rate (DCR). PFS and OS rates were comparable between the 2 groups. The median PFS in the mDCF and mDCX arms were 6.0 months (95% CI, 4.87–7.14) and 5.0 months (95% CI, 4.10–5.90) respectively (*P* = .08). The median OS in the mDCF and mDCX arms were 9.0 months (95% CI, 7.53–10.47) and 9.0 months (95% CI, 6.87–11.11) respectively (*P* = .07). Neutropenia, asthenia, stomatitis, and nausea/vomiting were the most frequently reported grade 3 to 4 adverse events (AEs). The rates of grade 3/4 AEs and dose reduction were comparable between the 2 groups. There was no treatment discontinuation due to grade 3 to 4 AE. As a first-line treatment for patients with mGC, mDCX and mDCF regimens have comparable efficacy and tolerability profiles.

## 1. Introduction

Gastric cancer remains one of the prevalent causes of cancer and cancer-related death worldwide.^[1]^ Despite many developments in diagnosis and treatment, the prognosis of metastatic or advanced gastric cancer is still not promising. The 5-year survival rate of gastric cancer is only 20%. Palliative systemic chemotherapy, targeted therapy, and immunotherapy are used to treat metastatic gastric cancer (mGC). Numerous genomic alterations can be found in gastric cancer, including mechanistic target of rapamycin, human epidermal growth factor receptor 2 (HER2), tyrosine-protein kinase Met (MET), epidermal growth factor receptor, fibroblast growth factor receptor-2, phosphoinositide 3-kinase, microsatellite instability, and claudin 18.2. Each gene has its peculiar pattern of genomic alterations.^[2]^

Combination chemotherapy is accepted as the standard first line treatment regimen for metastatic gastric cancer. In the treatment of mGC, docetaxel can be used as a single agent or in combination with cisplatin and 5-FU. 5-fluorouracil (5-FU) and its oral prodrug, capecitabine, are used sequentially. They block the enzyme thymidylate synthase, which is involved in DNA synthesis.^[3]^ Capacitabine has more tolerable toxicity profile than intravenous 5-FU. Changing 5-FU with capecitabine in Docetaxel-5-FU-cisplatin (DCF) regimen was aimed to keep efficacy meanwhile decreasing toxicity. Regimens should be chosen based on the patient’s performance status, medical comorbidities, and toxicity profiles.^[4]^ Three-drug (triplet) regimens are only recommended in select patients who are medically fit and have easy access to physicians and health care centers due to toxicity.^[4]^ The combination of FU and cisplatin was widely accepted as a safe and effective standard regimen, and studies focused on the benefits of adding a third agent, particularly a taxane, to this regimen.^[5]^

FOLFOX (5-FU, folinic acid, and oxaliplatin), XELOX (capecitabine and oxaliplatin), cisplatin/FU, capecitabine/FU, irinotecan, and taxan/platin combinations are the generally preferred regimens in the first -line setting of HER2-negative disease.^[6–10]^ The triplet combination of DCF chemotherapy regimen is an active and tolerable first-line treatment for patients with mGC.^[11]^ In the first-line treatment of mGC, the DCF regimen was found to be more effective than the cisplatin/FU/folinic acid regimen with an acceptable toxicity profile.^[12]^ After DCF chemotherapy, maintenance treatment with capecitabine was found to be safe, and this strategy can prolong the time to progression.^[13]^ The triplet regimen may increase R0 resection rates, DFS, and overall survival (OS) in patients; however, high-grade toxicities are anticipated more with triplets.^[14]^

According to the literature, a triplet regimen may be beneficial in patients with poorly differentiated adenocarcinoma and liver metastasis.^[15]^ Different triplet agents have been compared in the meta-analysis, but there is limited data regarding the comparison of DCF and docetaxel, cisplatin, and capecitabine (DCX).^[16]^ DCX was well tolerated by Japanese mGC patients, and patients with lymph node metastasis were converted to surgery.^[17]^ Finding the best benefit-to-risk ratio among various first-line platinum or FU-based regimens may help us in sequencing different regimens.

In this retrospective study, we aim to crosscheck the efficacy and safety of mDCF and mDCX regimens for first-line treatment in patients with mGC.

## 2. Material and methods

After receiving Istanbul University Ethics Committee’s approval, we reviewed 112 patients with pathologically confirmed HER2-negative mGC patients who had de novo metastatic disease or recurrence (local or distant) after curative surgery between December 2010 and February 2021. Demographic information including the age and gender of the patients were recorded. The primary tumor site (i.e., the gastroesophageal junction–cardia or stomach), stage at the time of diagnosis, metastatic sites, and differentiation (well/moderate or poor) of the primary tumor were extracted from the hospital cancer registry. Clinical staging was classified concerning the 8th edition of the AJCC Stage Classification for GC. Patients with < 18 years of age, double primary cancers, HER2-positive disease (immunohistochemistry [IHC] score 3 + or score 2 + and FISH-positive), and the presence of serious comorbid conditions were excluded from analyses.

The primary assessments included a comprehensive medical history, physical examination, complete blood count (CBC), and hepatic and renal function tests. The staging was performed by thoracic-abdominopelvic computed tomography or magnetic resonance imaging scans. Bone scintigraphy and brain magnetic resonance imaging scans were performed if clinically indicated. CBC and blood chemistry were monitored before each cycle. Moreover, CBC was analyzed weekly before the application of the chemotherapeutic regimen. After 2 courses of chemotherapy and at the end of treatment, imaging modalities were repeated to assess response.

The patients were distributed into 2 groups based on the chosen salvage regimens: those who received the mDCF protocol, which included 60-mg/m^2^ docetaxel and cisplatin 40 mg/m^2^ on day 1 and 600-mg/m^2^ 5-fluorouracil continuous infusion per day on days 1 to 5, every 3 weeks, and those who did not. Docetaxel 60 mg/m^2^ plus cisplatin 60 mg/m^2^ on day 1 was combined with capecitabine 650 mg/m^2^ on days 1 to 14, every 3 weeks, in patients receiving mDCX.

The primary endpoint was progression-free survival (PFS). Secondary endpoints included objective response ratio (ORR), disease control rate (DCR), and safety profiles. According to the Response Evaluation Criteria in Solid Tumors (RECIST 1.1), the response evaluations were divided into 4 subgroups: complete response (CR), partial response (PR), stable disease (SD), and progressive disease. A CR or PR was used to define the ORR. The DCR was ingenerated from CR, PR, and SD. Toxicities were graded from 1 to 4 during all visits using the National Cancer Institute Common Toxicity Criteria Version 3.0.

### 2.1. Statistical analysis

For all statistical analyses, IBM SPSS Statistics for Windows (Version 25.0; IBM Corp., Armonk, NY) was used. Numbers and percentages were used for descriptive statistics. The Chi-square test is used to calculate proportions in independent groups. As the normal distribution condition was met, numerical variables in the 2 independent groups were tested using the Student *t* test. The time from the beginning of treatment to the disease progression or the date of death or failure to follow up was defined as PFS. OS was determined as the time between starting mDCF or mDCX chemotherapy and the patient’s death or loss of follow-up. The patients’ dates of death were acquired from the death notification system of the Ministry of Health. The 95% confidence interval (CI) was used to testify the relationship between survival time and each independent factor. The survival rates were calculated using the Kaplan–Meier method. The log-rank test was used to assess the OS of subgroups. The alpha statistical significance level was set to *P* < .05.

## 3. Results

The cohort’s detailed demographic and clinical characteristics are summarized in Table 1. The median age, gender, ECOG PS (Eastern Cooperative Oncology Group Performance Status), primary tumor site, tumor differentiation, disease stage at diagnosis, site of metastasis, and curative or palliative resection surgery were similar between the 2 groups. When comparing mDCF (n = 69) and mDCX (n = 43), the most common metastasis site was distant lymph nodes (87% vs 79%), followed by the liver (51% vs 40%), peritoneum (44% vs 37%), and lung (11% vs 7%). The presence of signet-ring histology was more frequent in the mDCX group (56% vs 38%, *P* = .05). Treatment of primary tumors with perioperative radiotherapy and/or perioperative chemotherapy was more common in the mDCF group comparing the mDCX group (38% vs 19%, *P* = .02).

**Table 1:** Demographic and clinical characteristics of mGC patients.

Characteristics	mDCF n* *= 69, *n* (%)	mDCX n = 43, *n* (%)	*P* value
Age, median ± SD (min–max)	58 ± 13.1 (18–79)	60 ± 13.1 (21–84)	.34
Gender (male/female)	52 (75)/17 (25)	29 (67)/14 (33)	.39
ECOG performance score (0/1–2)	35 (51)/34 (49)	22 (51)/21 (49)	.96
Primary tumor site (GEJ†-cardia/stomach)	20 (29)/49 (71)	10 (23)/33 (77)	.66
Differentiation (well-moderate/poor)	31 (45)/38 (55)	15 (35)/28 (65)	.33
Histology (adenocancer/signet-ring cell)	34 (49)/26 (38)	19 (44)/24 (56)	**.05** *
Disease stage at diagnosis (2–3/4)	25 (36)/41 (59)	9 (21)/34 (79)	.07
Site of metastasis			
Non-distant lymph node‡/distant lymph node	8 (12)/60 (87)	8 (19)/34 (79)	.40
Primary therapy			
Surgery (yes/no)	26 (38)/38 (55)	18 (42)/24 (56)	.47
Perioperative chemotherapy and/or radiotherapy (yes/no)	26 (38)/38 (55)	8 (19)/34 (79)	**.02** *

mDCF = modified docetaxel, cisplatin, 5-FU, mDCX = modified docetaxel, cisplatin, and capecitabine, mGC = metastatic gastric cancer.

* Statistically significant.

† Gastroesophageal junction.

‡ Liver, lung, bone, and peritoneum.

The response to treatment is demonstrated in Table 2. The median follow-up of the patients was 11.7 months (range: 2–50), and all were deceased during follow-up. The median number of mDCF and mDCX were 6 (range: 3–10) and 5 (range: 3–9) cycles, respectively, with no significant difference between groups (*P* = .19). All patients were eligible for the evaluation of response. Although the CR rate was not statistically significant, it was higher in patients receiving mDCF than in those receiving mDCX (10% vs 2%), but ORR and DCR were not different between these 2 treatment arms.

**Table 2:** Antitumor efficacy analysis of patients with mGC received mDCF versus mDCX regimen.

Response to therapy	mDCF n = 69, *n* (%)	mDCX n = 43, n(%)	*P* value
Complete response	7 (10.1)	1 (2.3)	.15
Partial response	20 (29.0)	16 (37.2)	.41
Stable disease	20 (29.0)	15 (34.9)	.54
Progressive disease	22 (32.0)	11 (25.6)	.53
Objective response rate (ORR)	27 (39)	17 (40)	.97
Disease control rate (DCR)	47 (68)	32 (74)	.53

mDCF = modified docetaxel, cisplatin, 5-FU, mDCX = modified docetaxel, cisplatin, and capecitabine, mGC = metastatic gastric cancer.

**Figure 1.**
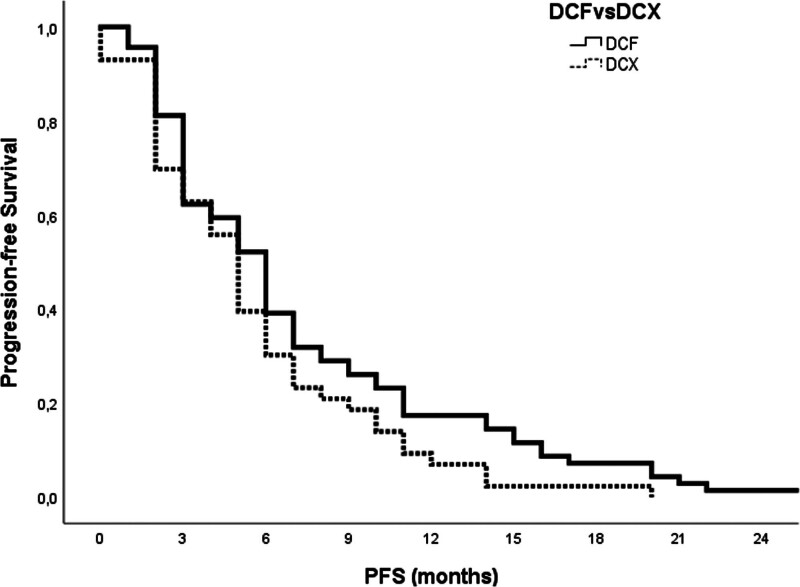
Progression-free survival curves in mGC patients based on 2 chemotherapy regimens (*P* = .08). mGC = metastatic gastric cancer.

**Figure 2.**
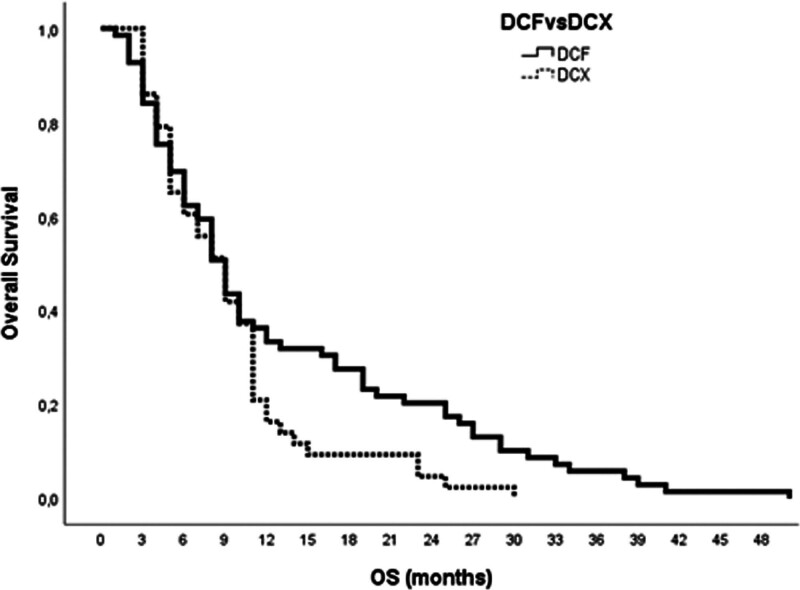
Overall survival curves in mGC patients based on 2 chemotherapy regimens (*P* = .07). mGC = metastatic gastric cancer.

The survival of mGC patients who received mDCF versus the mDCX regimen is depicted in Table 3. The median PFS was similar between the mDCF and the mDCX arms (6.0 months [95% CI, 4.9–7.1] vs 5.0 months [95% CI, 4.1–5.9], *P* = .08; Fig. 1). Neither OS was different statistically (mDCF: 9.0 months (95% CI, 7.5–10.5) vs mDCX: 9.0 months (95% CI, 6.9–11.1), *P* = .07; Fig. 2).

**Table 3:** Survival of patients with mGC received mDCF versus mDCX regimen.

Variables	mDCF (n = 69)	mDCX (n = 43)	*P* value
Progression-free survival			
Median survival (95% CI), months	6.0 (4.87–7.14)	5.0 (4.10–5.90)	.08
Overall survival			
Median survival (95% CI), months	9.0 (7.53–10.47)	9.0 (6.87–11.11)	.07

95% CI = 95% confidence interval, mDCF = modified docetaxel, cisplatin, 5-FU, mDCX = modified docetaxel, cisplatin, and capecitabine, mGC = metastatic gastric cancer.

Both regimens were well tolerated by the patients. Neutropenia (17% vs 20%), asthenia (14% vs 11%), stomatitis (12% vs 9%), and nausea/vomiting (10% vs 9%) were the most common grade 3 to 4 adverse events (AEs) in mDCF versus mDCX. The most common grade 3 to 4 hematological toxicity was neutropenia too. The rates of febrile neutropenia in mDCF and mDCX were 2% and 4%, respectively, and were successfully treated with supportive care, G-CSF (Granulocyte colony-stimulating factor), and antibiotics. There were no deaths as a result of neutropenic fever. If grade 3 to 4 toxicity occurred, secondary prophylaxis with G-CSF was administered until the end of treatment. In terms of grade 3/4 AE, there was no statistically significant difference between the groups. Hand–foot syndrome due to chemotherapy was observed frequently (mDCF: 40% vs mDCX: 45%), but only 7 (6%) patients had grade 3 to 4. In total twenty patients (mDCF: 12 patients vs mDCX: 8 patients) changed their regimen due to toxicity (14 patients [13%]) and noncompliance (6 patients [5%]), but treatment was not discontinued due to unacceptable grade 3 to 4 toxicity.

## 4. Discussion

Gastric cancer is a malignant condition with a generally poor outcome. There are dozens of cytotoxic agents which are used in treatment of mGC. Using a cytotoxic agent as monotherapy can be well tolerated but result in low ORR. Thus, various treatment approaches are being investigated, but studies comparing various chemotherapeutic regimens are rare. Triplet chemotherapies have more side effects, unless response rates are higher with these regimens.^[4,5,14]^ DCF combination chemotherapy is a standard and tolerable regimen for the first-line treatment of patients with mGC; however, DCX is another option for patients who do not tolerate 5-FU or do not wish to have a central port implanted.

In this retrospective study, DCF and DCX had similar PFS and OS in patients with untreated, HER2-negative, unresectable advanced gastric cancer. Cisplatin has long been utilized in the treatment of patients with gastric cancer. Doublets as first-line treatment for mGC have an ORR of 40%, a DCR of 84%, and a median PFS of 6.9 months in the literature.^[18]^

Moreover, triplet chemotherapy regimens are the fortitude of mGC. In the TAX325 trial, mOS was 9.2 months (95% CI, 8.4–10.6) and an mPFS was 5.6 months (95% CI, 4.9–5.9) in mDCF arm (4). In another study comparing mDCF and ECF regimens, in the mDCF arm mOS was 12.5 months (95% CI, 11.2–15.8), and mPFS was 7.5 months (95% CI, 6.2–9.7).^[19]^

A meta-analysis of 24 studies for evaluating the efficacy of the mDCF regimen in the first-line treatment of mGC showed that the mOS was 12.3 months (95% CI, 10.6–14.3), and the mPFS was 7.2 months (95% CI, 5.9–8.8).^[20]^ In our study, the median OS was 9.0 months (95% CI, 7.53–10.47) in the mDCF arm and 9.0 months (95% CI, 6.87–11.11) in the mDCX arm (*P* = .07). The mPFS in the mDCF arm was comparable with that of the TAX325 study and mDCF studies.^[4,20]^ In our study, the complete response rate in the mDCF arm (10%) was found to be numerically higher than that in the arm mDCX. However, the side effects were comparable in both groups.

There are some limitations in this study. The study was designed as a retrospective single-center study with a relatively small sample size. Nonetheless, it could serve as a clinical practice guide by presenting real-life data from patients with mGC receiving triplet chemotherapy.

More detailed randomized controlled prospective trials with large patient groups are required to provide more information on this topic.

## Author contributions

**Conceptualization:** Abdulmunir Azizy, Nail Paksoy, İzzet Doğan, Didem Tastekin.

**Data curation:** Nebi Serkan Demirci, Nail Paksoy, Didem Tastekin.

**Formal analysis:** Nebi Serkan Demirci, Nail Paksoy, Senem Karabulut, Didem Tastekin.

**Funding acquisition:** Nebi Serkan Demirci.

**Investigation:** Nebi Serkan Demirci, Nail Paksoy, İzzet Doğan, Senem Karabulut, Didem Tastekin.

**Methodology:** İzzet Doğan, Senem Karabulut, Didem Tastekin.

**Project administration:** Abdulmunir Azizy, Senem Karabulut, Didem Tastekin.

**Software:** Abdulmunir Azizy.

**Supervision:** Senem Karabulut, Didem Tastekin.

**Visualization:** Latif Karahan, Didem Tastekin.

**Writing – original draft:** İzzet Doğan, Latif Karahan, Didem Tastekin.

**Writing – review & editing:** Abdulmunir Azizy, Didem Tastekin.
